# Is Perioperative Antibiotic Prophylaxis Necessary for Pediatric and Adolescent ESIN Osteosyntheses?—A Retrospective Analysis of 939 Surgical Procedures

**DOI:** 10.3390/children13020168

**Published:** 2026-01-25

**Authors:** Lino Hundhausen, Christian Wulbrand, Eva Scheerer-Harbauer, Patrik Sperling, Clemens Memmel, Alexander Hanke

**Affiliations:** 1 Department of Pediatric Surgery and Pediatric Orthopedics, Clinic St. Hedwig, Barmherzige Brueder Regensburg, KUNO Pediatric University Medical Center, Steinmetzstraße 1-3, 93049 Regensburg, Germany; 2 Department of Trauma Surgery, University Medical Center Regensburg, 93053 Regensburg, Germany; 3 Department for Trauma, Orthopedics and Sports Medicine, Hospital Barmherzige Brüder Regensburg, Prüfeninger Straße 86, 93049 Regensburg, Germany

**Keywords:** fracture, pediatric, PAP, perioperative antibiotic prophylaxis, FRI, fracture related infection, SSI, surgical site infection

## Abstract

**Background:** Elastic stable intramedullary nailing (ESIN) is widely used in pediatric trauma surgery for benefits such as early limb loading, short hospital stays, and easy implant removal. Perioperative antibiotic prophylaxis (PAP) is used to reduce surgical site infections (SSIs). However, the necessity of PAP in minimally invasive pediatric procedures like ESIN remains unclear. **Methods:** This retrospective analysis reviewed all pediatric fractures treated with ESIN osteosynthesis at a pediatric trauma center over a time span of 10 years. Focus was set on the use of PAP during implantation and complications in the period between osteosynthesis and implant removal, which was used as follow-up. **Results:** Between January 2013 and December 2023, a total of 979 ESIN osteosyntheses were performed. In total, 4.1% were lost to follow-up resulting in 939 eligible cases. Complications occurred in 14.7% of all cases. However, complications such as wound healing disorders, wound infections, and osteomyelitis were rare, at 1.7%. Between the different subgroups regarding the application of PAP, type of reduction, openness of the fractures, or localization, no significant differences were found. **Conclusions:** Besides the most minor complications, ESIN osteosynthesis in children and adolescents is safe treatment. No increased risk for complications was observed when closed reduction was performed without the administration of PAP.

## 1. Introduction

In 1940, intramedullary nailing with steel nails for the treatment of long bone fractures was introduced [1]. Subsequent developments led to more elastic and pre-bent nails that did not require additional fixation at the insertion site [2]. With the switch to titanium for improved elasticity, this led to the concept of elastic intramedullary nailing (ESIN) in pediatric surgery in the 1980s [3,4]. Current practices of intramedullary nailing in children are based on this concept and are now well established in pediatric and adolescent trauma surgery. Studies have shown that the technique of elastic intramedullary fixation is superior to other surgical methods, including earlier loading of the limb, which allows for earlier functional follow-up treatment, reduced duration of hospitalization, reduced time of surgery, improved cosmetic outcomes, and easier implant removal [5,6].

Another well-established surgical concept is perioperative antibiotic prophylaxis (PAP), which has been shown to reduce surgical site infections (SSIs) [7]. According to the recently published German “Perioperative and Periinterventional Antibiotic Prophylaxis in Surgery” guidelines, PAP is generally indicated in trauma surgery for the surgical fixation of closed fractures of long bones, with 1st- or 2nd-generation cephalosporins being recommended [8].

However, pediatric guidelines are frequently extrapolated from adult recommendations and are often adapted primarily based on body weight [9,10]. There is evidence that guidelines in pediatric surgery are limited in methodological quality and require improvement [11,12]. Furthermore, there is a paucity of data on pediatric-specific guidelines in the context of trauma surgery in children and adolescents, particularly regarding the administration of PAP. Considering potential adverse effects and the development of antimicrobial resistance, the routine use of PAP should therefore be critically evaluated. Previous research suggests that PAP may not be necessary for clean and minimally invasive surgical procedures [13,14].

The objective of this retrospective study is to assess whether ESIN osteosynthesis without PAP is associated with higher complication rates, especially PAP-related complications such as SSIs, wound healing disorders, and osteomyelitis. To better contextualize the findings, an additional analysis of general complications was also performed.

## 2. Materials and Methods

All pediatric and adolescent fractures (0–18 years) that were treated with ESIN osteosynthesis at a level I trauma center between January 2013 and December 2023 were analyzed for this study. Data was obtained from the clinic’s Information Technology (IT) system (SAP GUI v. 7.6.7, SAP Deutschland SE & Co. KG, Walldorf, Germany) and digital archive (JiveX v. 5.4.0.4, Visus Health IT GmbH, Bochum, Germany). The fracture localization and fracture pattern (classified by the AO classification system [15]) as well as application of PAP were of special interest. The indication for PAP was made individually based on the surgeons’ preference. If administered, PAP was applied intravenously as a single shot up to 30 min prior to incision. If no contraindications existed, Cefuroxime adapted to the patients’ weight was the primary choice. Clindamycin was used when allergies were present. The same substances were used for open fractures and administered upfront intravenously in the emergency department and given for one to three days.

Implant removal was used as follow-up, as is usually done after fracture consolidation and healing. After this point, especially complications related to PAP or its omission should not occur anymore. It is unlikely that a longer follow-up period would add value to answering the study’s main question. Only existing data and documentation was used. Cases were only excluded if no postoperative information was available or if crucial information was missing.

All files and documentation of the included patients were analyzed, and the parameters listed in Table 1 obtained. Specifically, wound-healing disorders were classified as wound dehiscence, prolonged wound secretion, or a healing duration exceeding 14 days. If additional signs of infection, such as erythema, swelling, or fever, were documented that led to the prescription of oral antibiotics, the condition was classified as a wound infection. When radiological abnormalities were found that resulted in surgical treatment and microbiological detection of bacteria, the complication was defined as osteomyelitis.

Evaluation was conducted with statistical analysis software (SPSS v. 24.0, IBM SPSS Inc., Chicago, IL, USA). All data was tested for normal distribution with the Shapiro–Wilk test. Results of non-parametric data are depicted as median, 25% and 75% quantile and total range. The Fisher–Freeman–Halton exact test with Monte Carlo simulation for low sample sizes and the Mann–Whitney U test, described as median, 25% and 75% quartiles, were used to test non-parametric data. No parametric testing was needed. The significance level was set at *p* = 0.05.

## 3. Results

**Figure 1:**
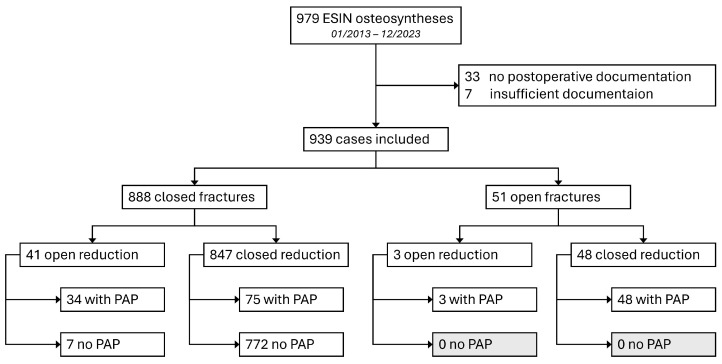
Flow chart of the distribution of included cases, fracture types, and application of PAP.

**Study population:** In total, 979 ESIN osteosyntheses were performed during the research period. Out of all 979 documented ESIN osteosyntheses, 33 cases (3.4%, 33/979) lacked documentation of postoperative examinations, and 7 cases (0.7%; 7/979) were excluded due to insufficient documentation. This resulted in a lost-to-follow-up rate of 4.1% (40/979) and 939 eligible cases (Figure 1).

In 20 patients (2.1%, 20/939), two ESIN osteosyntheses were performed at different times and different anatomic locations in separate surgeries. In one patient, two ESIN osteosyntheses were performed in the same session at different localizations. This results in a total number of 918 treated patients. Male patients accounted for 63.7% (m = 598; f = 341, ratio m:f = 1.8). The median age at time of surgery was 8.1 years [5.8 y; 11.5 y] (range 1–17 y). The median weight was 28 kg [20 kg; 41 kg] (range 7–90 kg). The median height was 137 cm [120 cm; 154 cm] (range 74–187 cm). However, this particular information was only documented in 55.2% of the cases (518/939).

**Figure 2:**
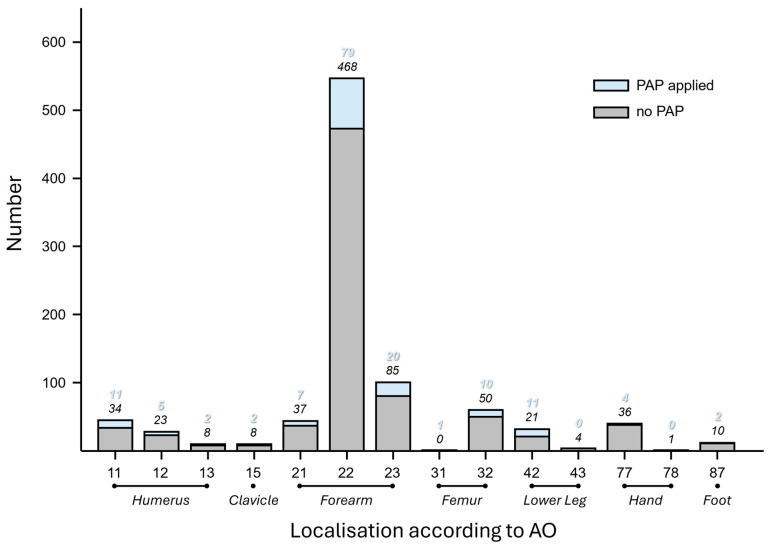
Histogram of fracture localization according to AO in the study population regarding application of PAP.

**Fracture Patterns:** In 55.0% (516/939) of all cases, the left side of the body was affected. The localization of the fracture was further classified according to the AO classification system [15]. By far, the most frequently affected region was the forearm shaft (AO 22), accounting for 58.3% (547/939) of all fractures, followed by fractures of the distal forearm (AO 23) with 11.2% (105/939), the femoral shaft (AO 32) with 6.4% (60/939), and the proximal humerus (AO 11) with 4.8% (45/939) (Figure 2).

The most common fracture types were diaphyseal forearm greenstick fractures (AO 22-D/2.1: 23.4%; 220/939), diaphyseal forearm transverse fractures (AO 22-D/4.1: 20.1%; 189/939) and complete metaphyseal fractures of the distal forearm (AO 23-M/3.1: 6.4%; 60/939).

Open fractures accounted for 5.4% (51/939) of all fractures. According to the Gustilo–Anderson classification system [16] 4.8% (45/51) were classified as 1st degree and 0.6% (6/939) as 2nd degree.

Furthermore, 10.8% (101/939) of the cases had a fracture-associated medical history: 5.9% (55/939) were treated surgically due to secondary dislocation after initial conservative treatment, 3.0% (28/939) were refractures after previous operative or conservative fracture treatment, 1.3% (12/939) were connected to a juvenile bone cyst, and 0.9% (8/939) to previous orthopedic surgery. Furthermore, 0.5% (5/939) had other risk factors in their medical history for fractures, such as osteogenesis imperfecta, developmental delay of unknown origin, increased tendency to fracture of unknown origin, and previous fracture consolidated in malalignment.

**Surgical technique:** In 62.7% (589/939) of the cases, surgery was performed on the same day the diagnosis was made, and in 22.2% (208/939), it was performed on the first day after. In 2.6% (24/939) of all cases, surgery took place two days after diagnosis, and in 12.6% (118/939) of the cases, more than two days elapsed between diagnosis and surgery [0 d; 1 d] (range 0–86 d).

In 3.8% (36/939) of the cases, K-wires were inserted in addition to the ESINs and buried below skin level. All of these were ulnar shaft fractures combined with a metaphyseal radial fracture.

The median duration of surgery was 29 min [19 min; 45 min] (range 4–219 min). There was no correlation between the duration of surgery and wound infection (*p* = 0.632) or wound healing disorders (*p* = 0.726).

Fracture reduction was performed in a closed manner in 95.3% (895/939) of the cases and open in 4.7% (44/939) of cases. In 85.4% (802/939), non-absorbable cutaneous sutures were used, while in the other 14.6% (137/939), absorbable sutures were used. No association between the type of suture and the occurrence of wound infection (*p* = 0.373) or wound healing disorder (*p* = 0.157) was evident.

**PAP:** Perioperative antibiotic prophylaxis was administered in 17.0% (160/939) of all ESIN osteosyntheses. Cefuroxime was given in 90.6% (145/160) of the cases at a median dose of 25.4 mg/kg [11.5 mg/kg; 48.2 mg/kg]. Clindamycin was used in 6.9% (11/160) of the cases at a median dose of 13.5 mg/kg [8.7 mg/kg; 16.9 mg/kg]. Ceftriaxone, ampicillin, and amoxycillin/clavulanic acid were each used only once. In one case, there was insufficient documentation of the type of PAP.

PAP was given in 84.0% (37/44) of the cases when open reduction was performed, compared to only 8.9% (75/847) during closed reduction. All 51 1st- or 2nd-degree open fractures received PAP regardless of the mode of reduction (see also Figure 1).

No side effects of PAP administration were documented in any case.

**Follow-up and complications:** In 934 cases, the ESINs were removed after a median of 105 days [90 d; 124 d] (range 4–1516 d). All implant removal procedures were performed under general anesthesia. It should be mentioned that in 3 cases (0.3%, 3/939), the material was only used as a reduction aid for radial head fractures, and not implanted, making removal unnecessary. In 2 cases (0.2%, 2/939), the implant was not removed because of medical reasons. These 5 cases had sufficient postoperative documentation longer than 105 days and were included in the follow-up. This resulted in 939 eligible cases.

A total of 165 (17.6%, 165/939) complications were documented in 138 cases (14.7%, 138/939) before implant removal. The most common complication was “sensory disturbance,” at 4.5% (42/939). The second most common was “refracture,” at 3.5% (33/939). Of those, 21 had a history of prior osteosynthesis of the same bone, while 12 were with ESINs still in situ.

The median age and weight of patients with complications were 9.9 years [6.7 y; 13.1 y] (range 1–17 y) and 36 kg [23 kg; 49 kg] (range 23–90 kg). Without complications, the median age and weight were significantly lower at 8.0 years [5.6 y; 11.2 y] (range 2–17 y) and 31 kg [20 kg; 40 kg] (range 7–88 kg] (*p* < 0.001, respectively). When considering only PAP-associated complications, no statistically significant differences were observed for age (*p* = 0.399) or weight (*p* = 0.571).

All complications listed by region are shown in Table 2. Complications listed by type of fracture, mode of reduction, and application of PAP are shown in Table 3.

Other minor deviations from the treatment path that were not classified as complications were documented. 15.7% (147/939) had limited terminal mobility compared to the uninjured side. 8.1% (76/939) developed pseudobursas or seromas around the tips of the ESINs found during implant removal. 2.8% (26/939) developed hypertrophic scars. 2.7% (25/939) had tolerable malalignment without further consequence. 2.7% (25/939) reported skin irritations caused by ESIN without sensory disturbance or perforation.

In one case, osteomyelitis occurred after closed reduction without using PAP (0.1%, 1/772). This case involved a 9-year-old boy with a closed forearm shaft greenstick fracture (AO: 22-D/2.1) on the left side. After closed reduction without PAP and the placement of two ESINs (2 × 2.5 mm), radiographic abnormalities were observed 6 weeks postoperatively. Consequently, early implant removal was performed along with intravenous and oral clindamycin administration. A staphylococcus aureus infection of the osteosynthesis material and bone was microbiologically confirmed. Two months after the implant removal, an elevated erythrocyte sedimentation rate was still present, and amoxicillin/clavulanic acid was prescribed orally. No further surgical interventions were needed, and the outcome was generally good despite load-related pain observed after 5 years.

## 4. Discussion

The main finding of this retrospective analysis is that pediatric and adolescent ESIN osteosyntheses are associated with a substantial complication rate. Nevertheless, infectious complications were rare and were not influenced by the administration of PAP.

**General complications:** The median age in this study was 8.1 years, with a male-to-female ratio of 1.7. Comparable findings have been reported in retrospective analyses of 553 pediatric forearm fractures (mean age 9.1 years, male-to-female ratio 1.8) [17] and 175 fractures (mean age 9.46 years, male-to-female ratio 1.6) [18]. A weight over 50 kg is known to be associated with higher complication rates [19]. In this analysis, older age and greater body weight were associated with an increased overall complication rate, reflecting the close correlation between these two variables.

Forearm fractures were by far the most prevalent fracture pattern in this study, accounting for almost three-quarters of all fractures (74,1%, 696/939), which aligns with the numerous studies found on this kind of fracture pattern and treatment [20].

A review by Poutoglidou et al. [21] on pediatric forearm fractures treated with ESIN, including 56 articles, demonstrated a high variability in complication rates, ranging from 8.9% to 69%. The main complications were associated with the radial ESIN entry point, such as skin irritations or lesions of the superficial radial nerve and re-fractures. PAP-related and infectious complications were not the focus of this review.

In this study, “sensory disturbance” was also the most common complication, occurring in 4.5% (42/939) of cases. However, only 9.5% (4/42) of these disturbances persisted after the removal of the ESIN and were managed conservatively. Reversible nerve lesions were also witnessed in other studies with comparable rates of 4.5% [22] in long bones, 2.5% [17] in forearm fractures, and 8.3% [23] for neurovascular complications in tibia fractures.

At 3.5% (33/939), refractures were the second most prevalent complication in this study. This rate is comparable to findings from other studies focusing on forearm fractures, which reported rates of about 5% [21,24,25].

The threat of perforation or actual perforation of the ESINs accounted for 2.8% (26/939) of reported complications. This was statistically significant regarding the location of the fractures, leading to rates of up to 30% (3/10) in clavicle, humeral, and femoral fractures. Furthermore, rates of 4.1 to 8.0% [26,27] were found for clavicle fractures, 5.7% [28] for humeral fractures, and 15.4–19.1% [29,30] for femoral fractures. The wide range of motion combined with little soft tissue coverage in these regions might explain these findings.

**PAP and Infection-Rates:** Complications related to PAP were not influenced by age or weight, likely due to the generally favorable healing capacity of children and adolescents. Therefore, the analysis of this cohort as a whole was considered justified.

In this study, wound healing disorders or SSIs of a closed fracture with ESINs were throughout lower than 1.3% disregarding the application of PAP. This is consistent with the occurrence of 0.95% (11/1156) fracture-related infections in a retrospective analysis of pediatric osteosyntheses in general by Hrubá et al. [31].

Wound healing-related or infectious complications for pediatric forearm fractures treated with ESIN were reported at rates of 0.5% (1/202) [24], 1.15% (2/173) [32], and 7.8% (7/90) [33]. According to a review including 10 studies about pediatric femoral fractures, a summed-up rate of 3.8% (10/266) was depicted [34]. A review of 28 studies concerning fractures of the pediatric tibia and lower leg found 2.3% (19/835) superficial and 1.0% (8/835) deep infections [35]. However, none of these studies mentioned the application or the omission of PAP. Other reviews claimed that infections after forearm ESINs are common but lacked in detail and did not provide information about PAP [36,37].

A single case of osteomyelitis occurred in the subgroup of closed reduction without PAP (1/772). As this was by far the largest subgroup in this study, it cannot be conclusively determined if a similarly sized subgroup with PAP would also experience such a complication. Fernandez et al. [17] described one case of osteomyelitis after a first-degree open fracture of the ulna in a retrospective analysis of 553 forearm fractures.

For open fractures, there are strong recommendations for early prophylactic administration of antibiotics [8,38,39], which was performed in all 51 cases of open fractures in this analysis. Infectious complications were not different from the results of closed fractures. Pandya et al. [40] compared 14 open and 12 closed pediatric tibial fractures, but no difference in the infection rates was observed when antibiotics were administered timely. However, the subgroups were small, and no information on the application of PAP in the closed fracture group was given.

Percutaneous K-wire fixation is a procedure with similar invasiveness to ESIN osteosyntheses and is commonly used in pediatric and adolescent fracture treatment. A review by Abul et al. [41] including 2316 patients from 4 retrospective cohort studies and one randomized controlled trial did not show any difference regarding SSIs depending on PAP application. No beneficial effects of PAP were found for pinning of supracondylar fractures in a retrospective analysis of 622 cases [42].

Formaini et al. [43] demonstrated no benefit of PAP for minimally invasive orthopedic surgery in a pediatric population in a retrospective analysis of 2330 cases, including some osteosyntheses by screw or K-wires.

In the adult population, a review of the literature on diaphyseal forearm fractures involving 9 studies showed an infection rate of 6.3% (15/238) when treated with ESINs [44]. Furthermore, adult nailing of long bones is associated with an infection rate of 7.9% [45] to 11.8% [46] even if PAP was administered. Nevertheless, infection rates in children and adults are not directly comparable [47].

Taking a more abstract approach to this topic, closed reduction and ESIN osteosyntheses can be classified as Class I/Clean by the Surgical Wound Classification [48]. In pediatric and adolescent populations, PAP is generally not recommended for SWC I procedures [13], even if ESIN osteosyntheses were not explicitly named.

In summary, this is, to the authors’ knowledge, the first study evaluating ESIN osteosyntheses with respect to PAP application. The findings support the assumption that PAP may not be beneficial in closed fractures of children and adolescents treated by closed reduction and ESINs. Infection rates do not appear to differ significantly with or without PAP. Given the low incidence of SSIs and the minimally invasive nature of the procedure, routine PAP administration in closed pediatric fractures appears clinically unwarranted.

**Limitations:** Although a large cohort was analyzed, the study is subject to limitations related to its retrospective, single-center design, resulting in uneven subgroup sizes that limited comparability. PAP-related complications were rare, reducing the statistical power of the results and limiting the feasibility of multivariable analyses, despite the large cohort. The quality of documentation was in general of a high and detailed level, although information loss due to insufficient documentation cannot be excluded. Because examinations were conducted by various clinicians without a standardized protocol, a potential classification bias in complication assessment cannot be excluded. Both pediatric and trauma surgeons participate in the surgical care of children and adolescents at our institution, procedures were performed by surgeons from both specialties. Therefore, potential bias in treatment strategy cannot be completely ruled out. Some decisions regarding PAP, even in the management of open fractures, were based on individual judgment, as no randomization or internal study protocol was used. Thus, a selection bias regarding the PAP and no PAP subgroups cannot be fully ruled out, limiting the reproducibility of the results. Some of the authors performed the surgeries themselves, although a relevant bias is considered unlikely. Nevertheless, given the low complication rate observed even in the absence of PAP, the dataset provides a robust foundation for drawing meaningful conclusions.

## 5. Conclusions

Despite the occurrence of mainly minor complications, ESIN osteosynthesis in children and adolescents is a safe and effective treatment. For closed reduction and ESIN osteosynthesis, no increased complication rates were observed in the absence of PAP. Therefore, PAP does not appear to be necessary for these procedures. Nevertheless, prospective and multicenter studies are required to establish evidence-based guidelines for PAP use in pediatric and adolescent ESIN osteosynthesis.

## Figures and Tables

**Table 1:** Collected parameters sorted by category.

Patient data	Gender
	Weight
	Height
Diagnosis	Date of accident
	Date of diagnosis
	Fracture classification (AO classification system^15^)
	Fracture associated medical history (e.g., secondary dislocation, re-fracture)
	Degree of open fracture (Gustilo–Anderson^16^)
Surgery	Surgery duration (min)
	PAP (type and dose in mg)
	Type of wound closure (adsorbable vs. non-adsorbable)
Follow-up	Date and indication of implant removal
	Complications until implant removal
PAP associated complications	Wound healing disorder
	Wound infection
	Osteomyelitis
Other complications	Threat of perforation
	Perforation
	Sensory disturbance
	Motor function disorder
	Paresis
	Refracture
	Revision
	Pseudarthrosis
	Prolonged pain/CRPS (Complex Regional Pain Syndrome)

**Table 2:** Complications and complication rate depending on localization according to AO, indicated as percentages and absolute numbers (n). Comparative statistics were carried out using the Fisher exact test with Monte Carlo simulation. Significant results are marked in red.

Region	Humerus			Clavicle	Forearm			Femur		Lower Leg		Hand		Foot		
AO	11	12	13	15	21	22	23	31	32	42	43	77	78	87	Sum % (n)	*p*
Number % (n)	**4.8 (45)**	**3.0 (28)**	**1.1 (10)**	**1.1 (10)**	**4.7 (44)**	**58.3 (547)**	**11.2 (105)**	**0.1 (1)**	**6.4 (60)**	**3.4 (32)**	**0.4 (4)**	**4.3 (40)**	**0.1 (1)**	**1.3 (12)**	**100.0 (939)**	
PAP-associated complications																
Wound healing disorder						0.6 (3)	1.0 (1)							8.3 (1)	**0.5 (5)**	0.411
Wound infection	2.2 (1)					0.9 (5)	1.9 (2)			3.1 (1)		2.5 (1)			**1.1 (10)**	0.576
Osteomyelitis						0.2 (1)									**0.1 (1)**	1.000
Other complications																
Threat of perforation		7.1 (2)		20.0 (2)					1.7 (1)			7.5 (3)			**0.9 (8)**	<0.001
Perforation	11.1 (5)	3.6 (1)		10.0 (1)	2.3 (1)	0.4 (2)	2.9 (3)		6.7 (4)	3.1 (1)					**1.9 (18)**	<0.001
Sensory disturbance		3.6 (1)		10.0 (1)	4.5 (2)	4.9 (27)			5.0 (3)	9.4 (3)	25.0 (1)	2.5 (1)		8.3 (1)	**4.3 (40)**	0.073
Motor function disorder						1.1 (6)						2.5 (1)		8.3 (1)	**0.9 (8)**	0.439
Paresis	2.2 (1)		10.0 (1)		4.5 (2)	0.4 (2)	1.0 (1)			3.1 (1)					**0.9 (8)**	0.038
Refracture		10.7 (3)	20.0 (2)			4.4 (24)	1.9 (2)		1.7 (1)			2.5 (1)			**3.5 (33)**	0.165
Revision	2.2 (1)	10.7 (3)			4.5 (2)	2.0 (11)	4.8 (5)	100.0 (1)	1.7 (1)	3.1 (1)				8.3 (1)	**2.7 (26)**	0.031
Pseudarthrosis						0.4 (2)			1.7 (1)						**0.3 (3)**	0.689
Prolonged pain/CRPS	6.7 (3)					0.2 (1)								8.3 (1)	**0.5 (5)**	0.010
Sum (n)	11	10	3	4	7	84	14	1	11	7	1	7	0	5	**17.6 (165)**	

**Table 3:** Complications and complication rate depending on surgical technique, indicated as absolute numbers (n) and percentages. Comparative statistics were carried out using the Fisher exact test with Monte Carlo simulation and indicated as *p*-values. Significant results are in red.

Group	Closed Fracture Open Reduction + PAP	Closed Fracture Open Reduction No PAP	Closed Fracture Closed Reduction + PAP	Closed Fracture Closed Reduction no PAP	Open Fracture Open Reduction + PAP	Open Fracture Closed Reduction + PAP	Sum % (n)	*p*
Number % (n)	3.6 (34)	0.8 (7)	8.0 (75)	82.2 (772)	0.3 (3)	5.1 (48)	100.0 (939)	
PAP-associated complications								
Wound healing disorder			1.3 (1)	0.4 (3)		2.1 (1)	0.5 (5)	0.242
Wound infection			1.3 (1)	1.2 (9)			1.1 (10)	0.860
Osteomyelitis				0.1 (1)			0.1 (1)	1.000
Other complications								
Threat of perforation	2.9 (1)		1.3 (1)	0.7 (5)		2.1 (1)	0.9 (8)	0.200
Perforation	2.9 (1)		6.7 (5)	1.3 (10)		4.2 (2)	1.9 (18)	0.027
Sensory disturbance	8.8 (3)	14.3 (1)	4.0 (3)	4.0 (31)	33.3 (1)	2.1 (1)	4.3 (40)	0.078
Motor function disorder				1.0 (8)			0.9 (8)	1.000
Paresis	5.9 (2)		1.3 (1)	0.5 (4)		2.1 (1)	0.9 (8)	0.036
Refracture	2.9 (1)		2.7 (2)	3.6 (28)		4.2 (2)	3.5 (33)	0.961
Revision	5.9 (2)		2.7 (2)	2.6 (20)	33.3 (1)	2.1 (1)	2.8 (26)	0.139
Pseudarthrosis	2.9 (1)	14.3 (1)		0.1 (1)			0.3 (3)	0.010
Prolonged pain/CRPS				0.7 (5)			0.5 (5)	1.000
Sum (n)	11	2	16	125	2	9	17.6 (165)	

## Data Availability

The original contributions presented in this study are included in the article. Further inquiries can be directed to the corresponding author.
